# Developing a Reference Database for Typical Body and Organ Growth of the Artificially Reared Pig as a Biomedical Research Model

**DOI:** 10.3389/fped.2021.746471

**Published:** 2021-12-01

**Authors:** Vinh H. Vu, Sharon M. Donovan, Lauren R. Brink, Qian Li, Gabriele Gross, Ryan N. Dilger, Stephen A. Fleming

**Affiliations:** ^1^Traverse Science, Champaign, IL, United States; ^2^Division of Nutritional Sciences, University of Illinois, Urbana, IL, United States; ^3^Department of Food Science and Human Nutrition, University of Illinois, Urbana, IL, United States; ^4^Medical and Scientific Affairs, Reckitt|Mead Johnson Nutrition Institute, Evansville, IN, United States; ^5^Medical and Scientific Affairs, Reckitt|Mead Johnson Nutrition Institute, Nijmegen, Netherlands; ^6^Piglet Nutrition and Cognition Laboratory, Department of Animal Sciences, University of Illinois, Urbana, IL, United States

**Keywords:** pig, database, early infancy development, research model, weight for age, reference database, pig reference weight for age, typical growth

## Abstract

**Objectives:** The pig is a common model utilized to support substantiation of novel bioactive components in infant formula. However, reference ranges for outcomes to determine safety are unclear. Our objective was to use historical data to objectively define typical body and organ growth metrics of the domesticated pig in research.

**Methods:** Twenty-two studies were compiled to assess typical growth of body and organ weights in young pigs. Metadata were organized to include milk replacer sources, bioactive components, sex, breed, source of herd, feeding regimen, and rearing environment. A combination of statistical models including simple linear regression and linear mixed effect models were used to assess typical growth patterns.

**Results:** Over 18,000 data points from 786 animals were available. In general, minimal differences in the growth of pigs who were male and female, artificially- or sow-reared, or fed *ad libitum*- or by scheduled-feeding, were observed in the first 30 days of life (*P* > 0.05). A weight-for-age chart from reference pigs was developed to compare body weights of pigs demonstrating growth characterized as accelerated, typical, reduced, and failure to thrive to illustrate effects of dietary interventions. Distributions of relative brain, liver, and intestine weights (as % of total body weight) were similar between rearing environments and sexes. An alternative bivariate level approach was utilized for the analysis of organ weights. This approach revealed significant biologically-relevant insights into how deficient diets can affect organ weight that a univariate level assessment of weight distribution was unable to detect.

**Conclusions:** Ultimately, these data can be used to better interpret whether bioactive ingredients tested in the pig model affect growth and development within typical reference values for pigs in the first 30 days of life.

## Introduction

The National Academy of Medicine, formerly named The Institute of Medicine (IOM), provided guidelines on considerations to make in the testing of safety and quality of ingredients in infant formula (1). They stated that preclinical studies are a necessary component of safety testing, but that rodent models were not recommended due to the difficulty of conducting nutritional studies on pre-weaning rodents. The IOM recommended non-human primates and young pigs as alternative developmental models to pre-weaning rodents (1). Similarly, the European Food and Safety Administration (EFSA) provided guidance promoting the use of a neonatal pig model for a repeated dose study with direct oral administration for the study of non-absorbed substances intentionally added to foods for infants below 16 weeks of age (2). Similarities to the human in neuroanatomy (3), nutritional requirements (4), and gastrointestinal physiology (5) make the pig an ideal model for testing safety and efficacy of nutritional innovations. The artificially-reared pig is a common model utilized in pediatric nutrition research as it allows for control of nutritive intake when testing the effects of various dietary ingredients. Examples of this model in the literature include assessments of the safety and efficacy of milk fat globule membrane (6), soy- vs. dairy-based formulas (7), human milk oligosaccharides (8), and fortification of donor milk for preterm models (9).

Clinical trials that investigate novel formulas rely on standardized growth charts in order to assess growth and tolerance. Reference ranges are currently not readily available for pigs, which may lead to misinterpretation of data. Interpretation of research in the pig may be assisted with the creation of historical reference ranges for multiple biological parameters (e.g., body growth rates, circulating metabolite concentrations, hormone levels, histology, etc.). Outcomes such as growth (10), sow milk composition (4), neuroanatomy (11), and microbiome composition (5) have been described previously. However, numerous resources must be assembled to generate reference ranges, and we are unaware of meta-analyses describing the distribution of values for a given outcome. Systematic literature review and meta-analysis may be helpful to overcome these issues and build an understanding around a body of literature.

Thus, we propose the creation of a continuously updated database containing raw data on pig body and organ growth values. Here, we describe an initial step toward this goal by compiling raw data on body and organ weights from birth to 30 days postnatal (PND) along with metadata from rearing conditions across a decade of research. In particular, we assessed how rearing conditions, sex, and feeding regimens affected growth, as well as established reference distributions for both body and organ weights. Ultimately, we call for collaboration across public and private organizations to contribute to this database to achieve the common goal of improving the applicability of young pig models for the assessment of safety and efficacy of pediatric nutrition products.

## Methods

### Data Cleaning and Import for Database Construction

Data were collected from 22 previously performed studies investigating the role of early life nutrition in pigs conducted over 10 years at the University of Illinois, Urbana Champaign, 15 of which were published (6, 12–25) (Table 1). Data were retrieved either directly from publications, direct correspondence with the principal investigator of each study, or internal archives. The raw files from each study were reviewed for accuracy and completeness. Where relevant, paper records were reviewed against digital copies to verify accuracy. The reviewed raw data for each study were then converted into a common format (units of measurement, study and subject identifiers, study methodology, etc.) and used to construct a master database. This process was completed in R 3.6.2 (28) using the tidyverse 1.3.0 package (29). In doing so, the master database contained ~18,000 data points collected from 786 animals.

**Table 1:** Studies included.

**Entry**	**Study description**	**Rearing environment**	**Sex**	**Feeding regimen**	**Organs available**	**References**
1	Feeding trial on arginine supplementation	Sow-reared	MF	NA	-	(23)
2	Feeding trial on perinatal choline deficiency and metabolomics	Sow-reared	MF	NA	Brain, liver	(21)
3	Feeding trial on perinatal choline deficiency and brain development	Sow-reared	MF	NA	Brain, liver	(26)
4	Feeding trial on prebiotics and milk bioactive supplementation	Artificial	M	Scheduled	Brain, intestines	(17)
5	Study on choline deficiency and porcine milk composition	Sow-reared	MF	NA	-	(22)
6	Development of behavioral testing methods	Artificial	F	Scheduled	-	Unpublished
7	Feeding trial on alpha-lipoic acid supplementation	Artificial	M	Scheduled	Brain, intestine	(14)
8	Feeding trial on sialyllactose supplementation	Artificial	M	Scheduled	Intestine	(18)
9	Internal study comparing commercial milk replacers	Artificial	F	Scheduled	-	Unpublished
10	Feeding trial on pectin supplementation	Artificial	MF	Scheduled	-	(15)
11	Feeding trial on polydextrose and galactooligosaccharide supplementation	Artificial	M	Scheduled	Intestine	(16)
12	Internal study assessing pig growth in early life	Artificial	M	Scheduled	-	Unpublished
13	Feeding trial on iron deficiency	Artificial	M	*ad libitum*	Brain, intestine, liver	(20)
14	Internal study testing behavioral paradigms with sow-reared pigs	Sow-reared	MF	NA	-	Unpublished
15	Feeding trial on sialyllactose supplementation	Artificial	M	*ad libitum*	-	(24)
16	Study on brain development	Artificial and Sow-reared	M	*ad libitum*	Brain	(27)
17	Feeding trial on milk fat globule membrane supplementation	Artificial	M	*ad libitum*	-	(6)
18	Internal study on maternal egg intake and offspring behavior	Sow-reared	MF	NA	-	Unpublished
19	Feeding trial on polydextrose and galactooligosaccharide supplementation	Artificial	Not specified	Scheduled	Intestine	(12)
20	Feeding trial on phthalate exposure using di(2-ethylhexyl) phthalate (DEHP)	Artificial	MF	NA	Brain, intestine, liver, lung, heart, kidney, spleen	Unpublished
21	Feeding trial on polydextrose and galactooligosaccharide supplementation	Artificial	MF	Scheduled	Intestine	(13)
22	Study on immune system development in sow-reared pigs	Sow-reared	MF	NA	Intestine	Unpublished

This database is open source and is hosted on the Traverse Science GitHub repository. Here, in-depth documentation on the study database, variables collected, treatment of the data, and study methods and design are available. The goal of this initial data collection and analysis is to not only provide a reference growth chart based on the dataset included, but to also create a foundational database that is dynamic and open to allow researchers from other institutions to contribute their data. The metadata collected (Table 2) included sex, rearing environment, types of diet, organ weights (brain, intestine, liver, etc.), feeding regimens, institutions, facilities, and housing conditions, such as light cycles and feeding related parameters.

**Table 2:** Sample metadata captured^a^.

**Item**	**Description**
Investigator	Last name of principal investigator of the study
Institution	Institution where research was performed
Rearing environment	Whether pigs were reared artificially or by the sow
Sex	Male or female
Stocking density	Number of pigs per cage/pen/unit
Light cycle	Number of hours of daylight provided
Light cycle start	Hour of the day lights were turned on
Light cycle end	Hour of the day lights were turned off
Diet code	Internal descriptor to denote dietary group
Diet name	Name of dietary treatment
Antibiotic-use	Name of injectable antibiotics used
Iron dextran	Description of the use of iron dextran shots
Source herd	The name of the farm animals were sourced from
Genetics	Name of the breed used
Electrolytes	Description of how electrolyte supplementation was used
Castration status	Description of whether male pigs were left intact or castrated
Delivery	Description of the birth delivery method
Rearing description	Long-text format description of rearing conditions
Feeding description	Long-text format description of feeding regimen
Feed Form	Description of the form of feed
Feed Base	Description of the base formulation of the diet
Water, %	% of water content of reconstituted milk replacer
Feed rate, g milk / kg bw	Amount of milk provided
Feeds per day	Number of times pigs were fed per day
Feed interval, hours	Number of hours between feeds
Feed start	Hour of the day feeding began
Feed end	Hour of the feeding ended
Publication URL	URL to the study publication

a *A more detailed list and description of metadata are available in a public repository accessible at [https://github.com/Traverse-Science/Pig-database]*.

### Inclusion/Exclusion Criteria and Reference Group Definition

**Figure 1:**
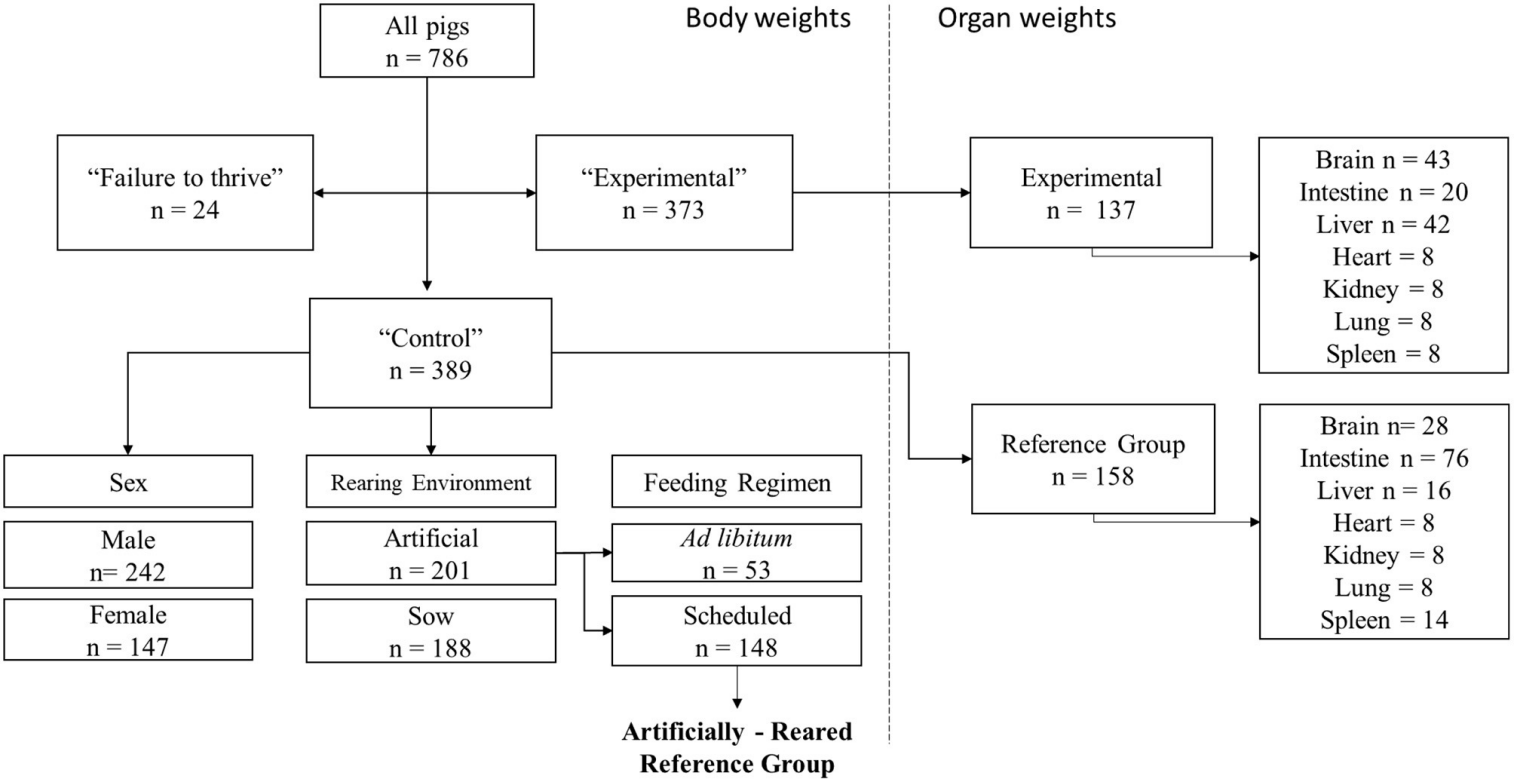
Schematic for the different inclusion and exclusion categories for database construction. Data from all 786 pigs available in total were separated into three main categories consisting of failure to thrive, Control, and Experimental groups. Control and Experimental pigs represent those fed a control or experimental diet in their respective study. Control pigs were then subcategorized by sex, rearing, and feed styles. Artifically-Reared pigs fed in a scheduled manner were then selected as reference group for assessment of typical body weight growth. Available organ weights from Control and Experimental groups were also then captured. Samples were pooled together, independent of sex, rearing environment, and feeding regimen.

A group of pigs to serve as the reference group was created by establishing the following inclusion criteria. First, pigs were categorized into two main groups: Control and Experimental (Figure 1). Control pigs were those from any study fed a respective control diet formulated to be nutritionally adequate, and Experimental pigs were those fed any experimental diet, typically in context of the addition or deficiency of a nutrient of interest. A group denoted as “failure to thrive” (FTT) was included in its own category. These pigs were labeled as FTT if their body weight did not increase within a 1- to 2-week period from PND 2 (the most common study enrollment date), which resulted in those subjects being humanely euthanized. Pigs with known medical conditions without an impact on growth (e.g., clinically diagnosed infections) that were removed from a study were not labeled as FTT but were included in the database. Pigs in the Control category were further sub-categorized based on sex (female vs. male), rearing environment [artificially-reared (AR), vs. sow-reared (SR)] and feeding regimen (scheduled vs. *ad libitum*). AR pigs separated by feeding regimen, and pigs fed in a scheduled (during a discrete time of day) manner were retained in the final AR Reference group. Growth from the AR Reference group was considered “typical,” and growth of pigs from Experimental groups was compared to this reference. For assessing organ weight, pigs that were fed Control diets were included in a separate reference group for organ weights. Due to limited availability of data, organ weight data was not stratified by sex, rearing environment, or feeding regimen.

### Statistical Analysis

To visualize the data and plot model fit, a polynomial regression model with non-linear covariates was employed. Polynomial regression is a form of regression analysis that explores the relationship between the independent and dependent variables and models them as nth degree polynomials of the independent variable (30). Such a model for a single predictor, *X*, is:


(1) $$\begin{array}{r} Y=\;\beta_{0}\;+\beta_{1}X+\beta_{2}X^{2}+\;\ldots +\beta_{h}X^{h}+\;\epsilon \end{array}$$

Here *h* is the degree of the polynomial. For lower degrees, the relationship has a specific name associated with it (e.g., *h* = 2 is called quadratic, *h* = 3 is called cubic, *h* = 4 is called quartic, etc.). *Y* is the response variable and *X* is the predictor variable. Here, *Y* is body weight, *X* is PND, and *h* is 3. The degree of polynomial (h) was initially set as quadratic (Eq. 1); however, after analysis, there were clear signs in the residuals and normal probability plot (i.e., quantile-quantile or Q-Q plot), suggesting that a higher-order model was needed. Refining the polynomial degree to *h* = *3* (cubic) yielded better uniform randomness in the residuals, and a normal probability plot in which the points did not deviate from the projected straight line, suggesting a normal distribution.

For the primary aim, the effect of PND as well as sex, rearing, and feeding regimen on the body weight of pigs were examined via mixed effect model analysis in R using the lme4 package (31). The approach to use a mixed-effects model rather than repeated measures methods such as analysis of variance (ANOVA) was selected, because the mixed-effect modeling allows for better handling of dependencies in repeated measures data (32). Individual pigs were treated as random effects. The predictors of interest (age, rearing environment, sex, and feeding regimen) were entered as main effects. Therefore, our model consisted of both fixed and random effects. In the initial step, subjects were entered as a random factor nested within study and study as its own error term. Then the main predictors were tested: age (PND), experimental (reference or experimental diets), sex (male or female), rearing environment (artificial vs. sow rear), and feeding regimen (*ad libitum* or scheduled).

Quantile regression was used to generate the growth percentile curves for the AR Reference group. In this model, quantile regression was used to calculate the quantile (percentage) for a particular value in the feature variables. Similar to linear regression, the beta coefficients (linear effect parameters) became functions with a dependency of the quantile. Finding values for the beta estimates at a particular quantile value is the same process as is for standard linear calculation, except the median absolute deviation is used and not the mean. Kernel density plots for visualizing the organ weight distribution were charted using the ggplot2 (33) package in R.

Statistical analysis comparing organ weights of the reference group to select Experimental groups (choline deficient, iron deficient, iron sufficient, and di(2-ethylhexyl) phthalate (DEHP) were completed in R using the student's *t*-test. All groups were compared to the reference group. Statistically significant differences between groups are visualized directly in boxplots with the following labels: ns (non-significant) *P* > *0.05*, ^*^P ≤ 0.05, ^**^P ≤ 0.01, ^***^P ≤ 0.001, ^****^P ≤ 0.0001. Analysis of slope was done by computing the coefficient of the interaction term for each regression. The ANOVA *p*-value from the interaction of body weight by organ weight (relative and absolute), was then examined and pairwise analysis was carried out to compare the different groups for statistical significance.

## Results

### Data Availability

In total, data from 786 pigs were retrieved for analysis (Figure 1, left panel). The Control groups and Experimental groups contained 389 and 373 animals, respectively. The Control group was further categorized into main effects of sex (n_male_ = 242, n_female_ = 147), rearing environment (n_artificial_ = 201, n_sow_ = 188), and feeding regimen (n_adlibitum_ = 53, n_Scheduled_ = 148). Data on organ (brain, intestine, liver, heart, kidney, lung, and spleen) weights were also included (Figure 1, right panel) for 158 animals in the reference group and 137 animals in the Experimental group. Due to high variation, unbalanced data, and multicollinearity, covariates such as breed, source of herd, dose of milk provided, and nutritional composition other than specific dietary interventions were not included in the analyses.

### Body Weight

**Figure 2:**
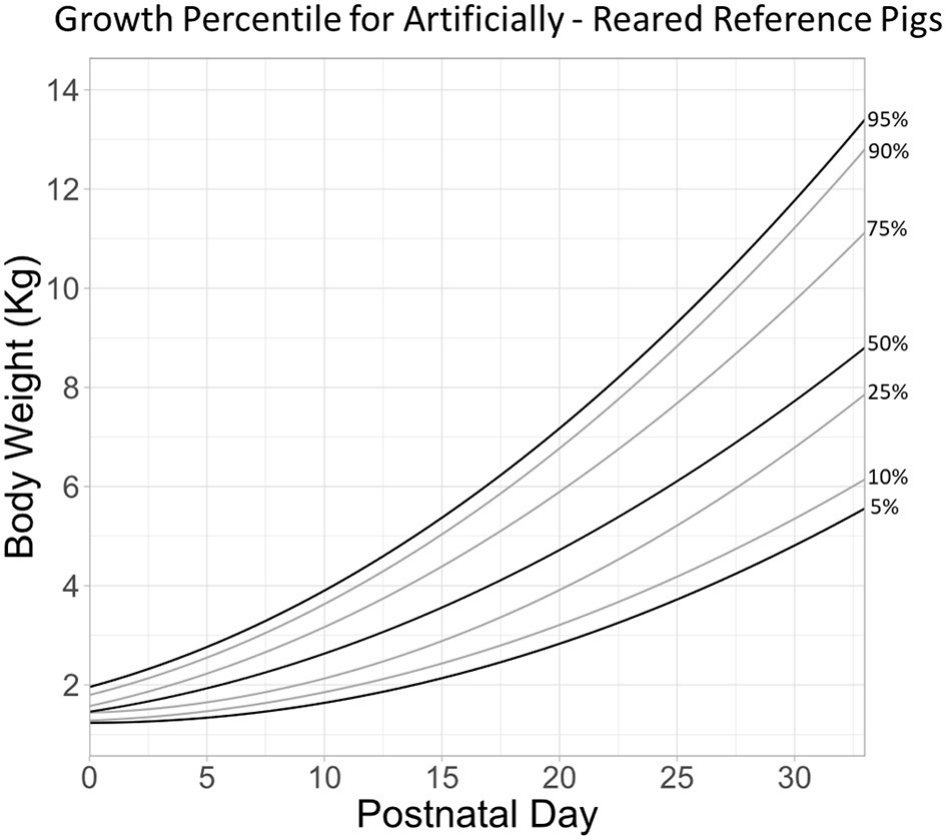
Growth percentile chart for artificially reared reference pigs is plotted as body weight as a function of postnatal day. Lines are growth quantile fits corresponding to 0.05, 0.10, 0.25, 0.50, 0.75, 0.90, and 0.95 quantiles.

**Figure 3:**
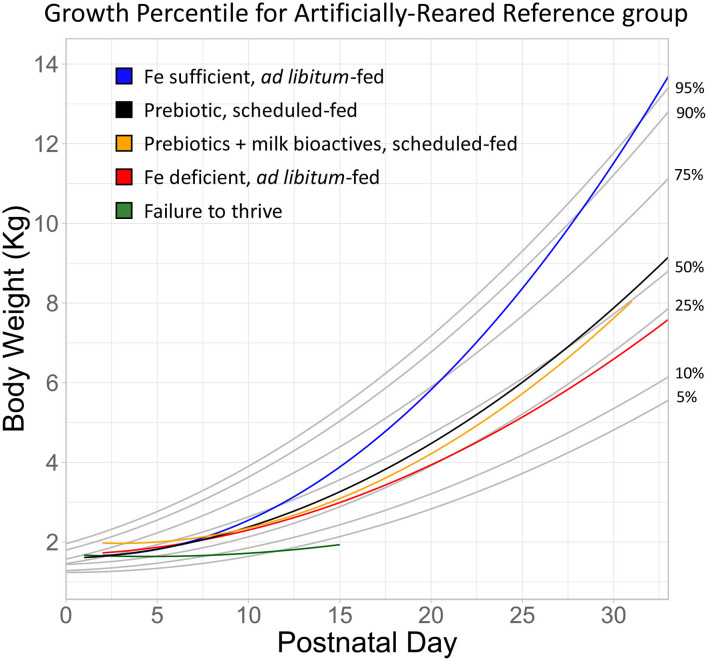
Growth of artifically-reared pigs from representative experimental groups overlayed onto growth curves developed from the Artificially Reared Reference group animals. The blue and red lines represent *ad libitum*-fed pigs on an iron sufficient or deficient diet (20). The black line represents scheduled-fed pigs supplemented with a prebiotic combination (polydextrose and galactooligosaccharide) (16). The yellow line represents scheduled defined-fed pigs supplemented with prebiotics and milk bioactives (17). Lastly, the green line represents pigs that exhibited failure to thrive. The body weight of these pigs did not increase within two weeks of study enrollment, resulting in humane euthanasia. All together, the plotted cases represent accelerated growth (blue), typical growth (black and yellow), reduced growth (red), and failure to thrive (green).

**Figure 4:**
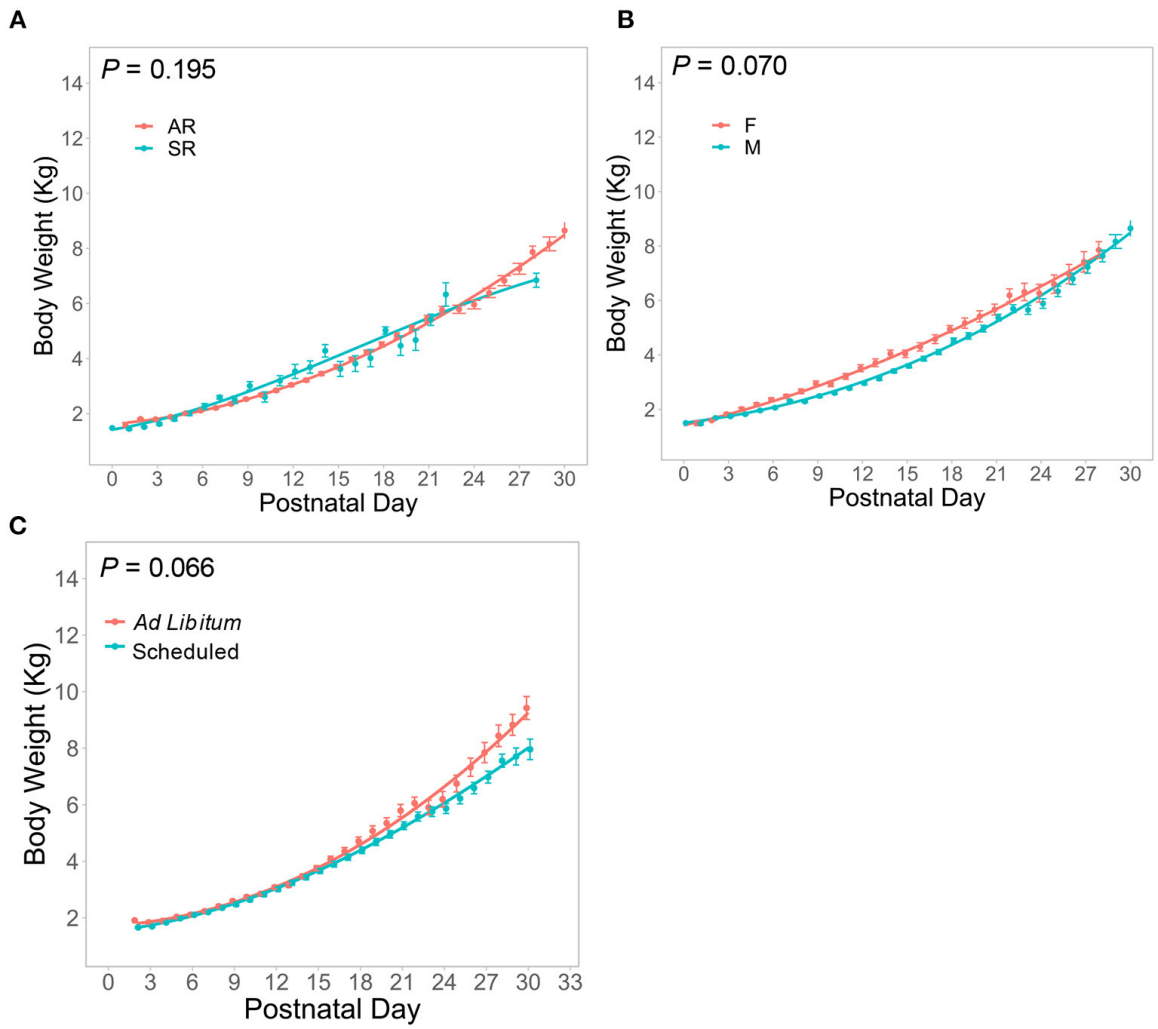
Body Weight data as a function of PND from the Control group are plotted for **(A)** artificially reared (AR) and sow-reared (SR) pigs, **(B)** female (F) and male (M) pigs, and **(C)** *ad libitum-* and scheduled-fed pigs, demonstrating very similar growth patterns across these groups between PND 0 and 30.

A pig growth percentile plot (Figure 2) was generated from the AR Reference group (n = 148) fed according to a defined pattern. The growth percentile plot is divided into seven growth quantiles (0.05, 0.10, 0.25, 0.50, 0.75, 0.90, and 0.95) to match the style used by the World Health Organization to track weight-for-age scores in human infants (34). In order to demonstrate experimental variation, select groups from the Experimental or FTT animals were chosen to exemplify exceptionally high growth [*ad libitum-*fed pigs from Knight et al. (20)], typical growth (pigs fed prebiotics with added milk bioactives such as lactoferrin and the milk fat globule membrane), reduced growth [*ad libitum*-fed iron deficient pigs from Knight et al. (20)], and severely poor growth (FTT) compared to growth of the AR Reference group plotted in the graph (Figure 3). Despite slightly different trajectories between groups, effects of rearing environment (Figure 4A*, P* = 0.195), sex (Figure 4B, *P* = 0.070), and feeding regimen (Figure 4C, *P* = 0.066) on body weight were found to be minimal.

### Organ Weight

**Figure 5:**
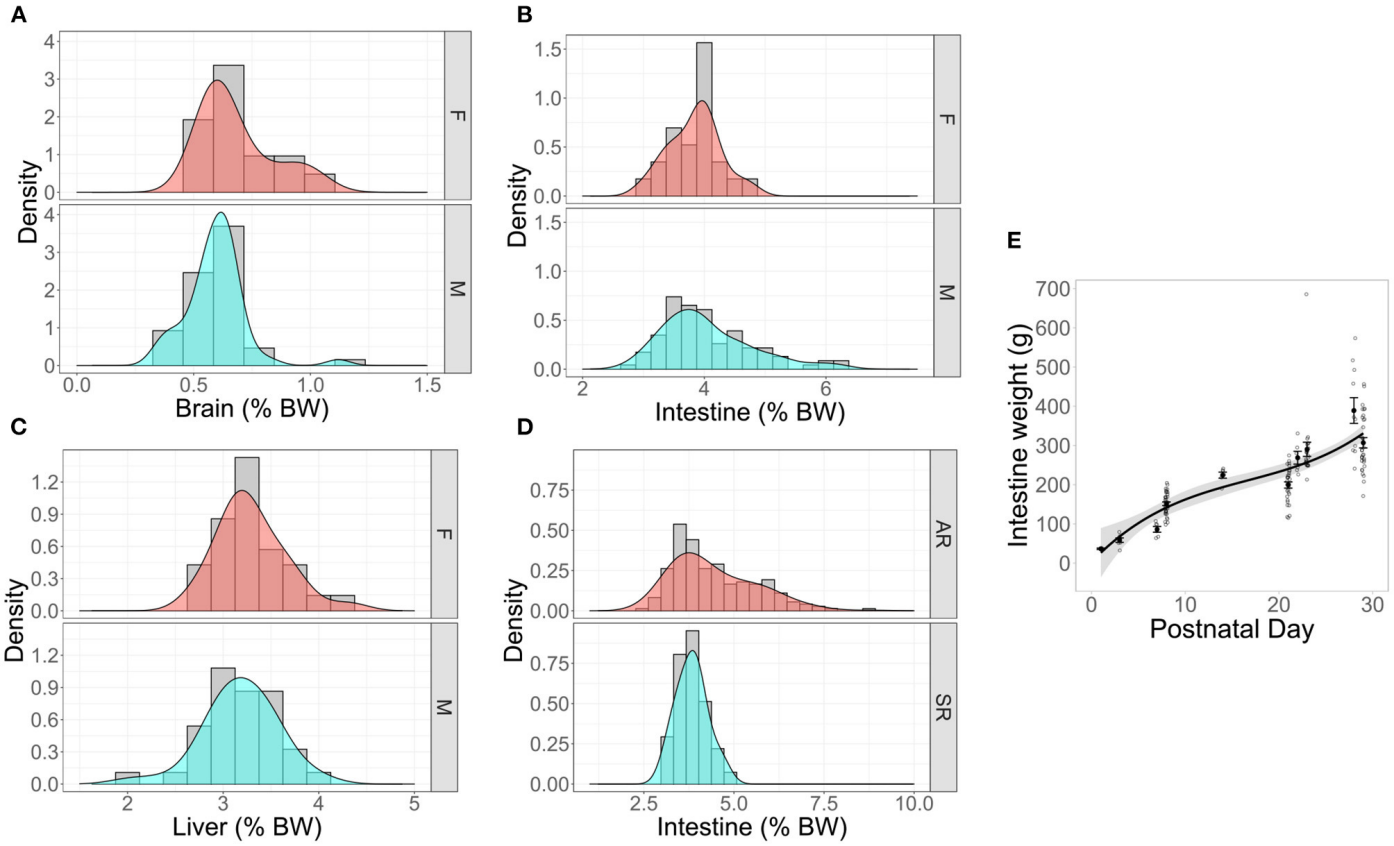
Density plots with overlayed histograms showing smoothed distributions of organ weights of reference pigs stratified by **(A–C)** sex or **(D)** rearing environment. “Density” represents the function used to calculate the probability of getting a value between a range of values on the x-axis. The peaks of the density depict where values are concentrated over a given interval. The density plotted as percent of total body weight (BW) for **(A)** brain, n_male_ = 20, n_female_ = 8, **(B)** intestines n_male_ = 60, n_female_ = 15, and **(C)** liver n_male_ =8, n_female_ = 8. **(D)** A density plot of percent intestinal growth in artifically-reared (AR) and sow-reared (SR) artificial reference pigs, n_AR_ = 57, n_SR_ =18. **(E)** Absolute intestinal weight plotted as a function of PND (PND 1, n_sample_ = 3; PND 3, n_sample_ = 7; PND 7, n_sample_ = 6; PND 8, n_sample_ = 34; PND 14, n_sample_ = 6; PND 21, n_sample_ = 31; PND 22, n_sample_ = 7; PND 23, n_sample_ = 21; PND 28, n_sample_ = 11; PND 29, n_sample_ = 31) in artificially-reared reference animals indicates linear increase over time.

Organ weights were normalized to body weight at the time of collection (PND 20–32) and are expressed as percentages of total body weight for the brain, liver, and intestine (from the duodenum to the ileum). They are shown for Control pigs by sex (Figures 5A–C), and for intestinal growth by rearing environment (Figure 5D). Intestinal weight as a percentage of body weight stratified by rearing environment revealed that AR pigs exhibited higher variability in the distribution of their intestinal weight compared to SR pigs (Figure 5D). The distributions of relative organ weights between males and females were almost entirely overlapping. Intestinal weight linearly increased with PND during the first 30 days of growth (Figure 5E).

**Figure 6:**
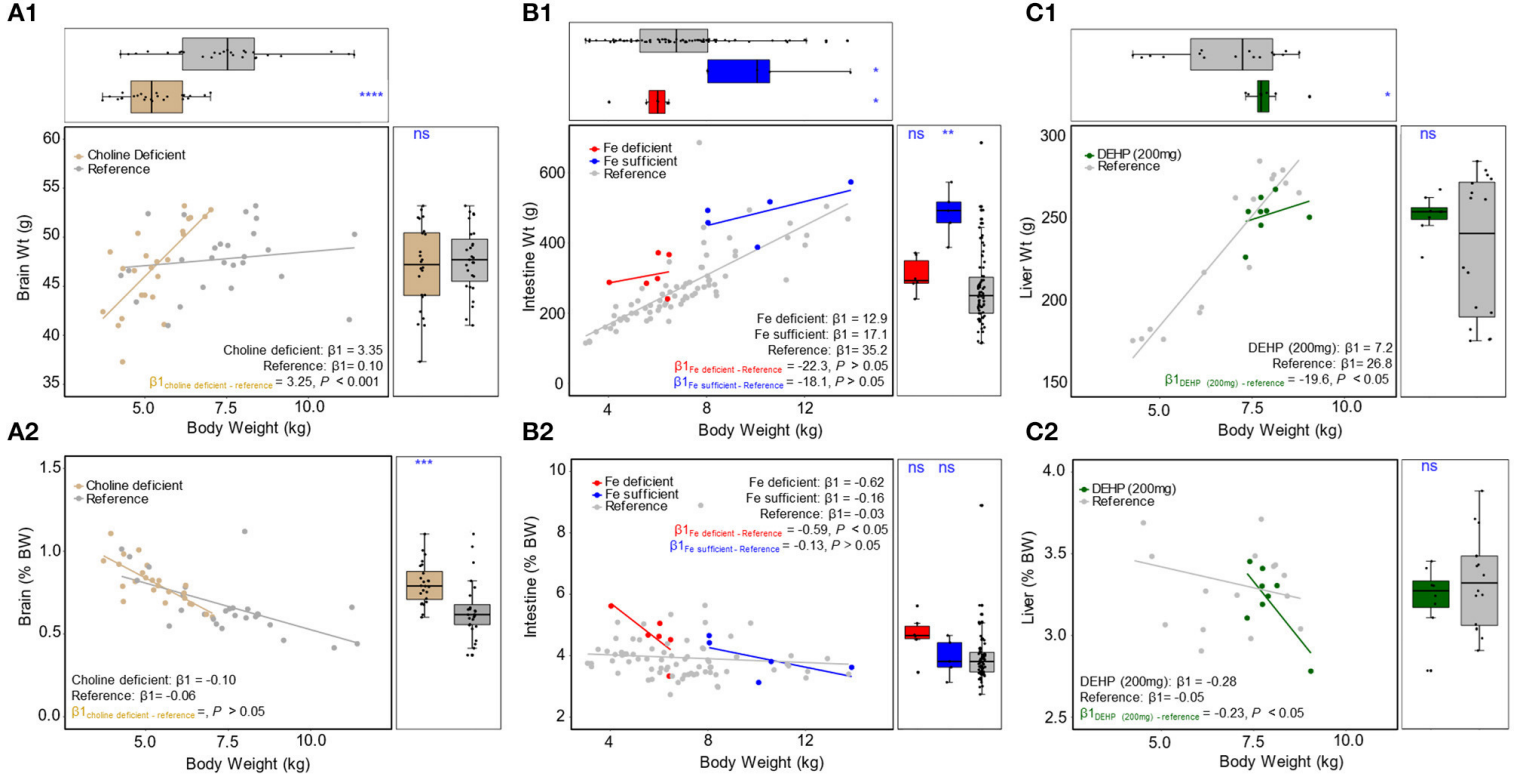
Scatter plot with marginal boxplots of absolute **(top)** and relative **(bottom)** organ weights are plotted as a function of BW for **(A1,2)** brain, comparing choline deficient experimental pigs and typical reference pigs, **(B1,2)** intestine, comparing iron deficient, iron sufficient, and typical reference pigs, and **(C1,2)** liver, comparing DEHP (200 mg) and typical reference pigs. ns *p* > *0.05*, *p ≤ 0.05, **p ≤ 0.01, ***p ≤ 0.001, ****p ≤ 0.0001.

Given similarity in organ weight distributions between rearing environments and sex, data on organ weights from all Control pigs were pooled to create a reference group for brain, intestine, and liver weight. This reference group was then compared on an absolute, relative, and bivariate (with body weight regressed against absolute or relative organ weight) basis to select variables within Experimental groups known to alter growth (e.g., choline deficiency, iron deficiency, and di(2-ethylhexyl)phthalate (DEHP) exposure, Figure 6). Choline deficient pigs exhibited similar absolute brain weight (Figure 6A1, *P* > 0.05), but higher relative brain weights compared to the reference group (Figure 6A2, *P* ≤ 0.001). Absolute and relative intestinal weights were not different between iron deficient and reference groups (Figures 6B1,2, *P* > 0.05), and this was also the case for absolute and relative liver weights between DEHP-fed pigs and the reference group (Figures 6C1,2, *P* < 0.05).

Across all instances, the relationship between body weight and absolute/relative organ weight (assessed via regression) revealed patterns suggesting atypical organ weight in animals fed diets known to alter development. Analysis of the slopes for brain weight regressed against body weight showed a significant difference between choline deficient pigs and reference pigs for the absolute brain weight (Figure 6A1, *P* <0.001), but not relative brain weight (Figure 6A2, *P* > 0.05). Slope analysis did not reveal a significant difference when comparing iron deficient pigs to reference pigs for body weight regressed against absolute intestine weight (Figure 6B1, *P* > 0.05). There was a statistically significant difference between slopes on a relative scale (Figure 6B2, *P* < 0.05) for the iron deficient group. When comparing the slopes of iron sufficient pigs to reference pigs, there were no differences found (Figures 6B1,2, *P* > 0.05) on either an absolute or relative scale. For DEHP-fed pigs, slope analysis revealed significant differences in liver weight regressed against body weight as compared to reference pigs (Figures 6C1,2, *P* < 0.05) on both an absolute and relative scale.

## Discussion

The database described here is the only known source of compiled data on body and organ weights in young AR pigs raised in varying environments from studies conducted over a 10-year period. This work represents the first step toward creating a unified source of biological data on the use of the domestic pig as a biomedical research model. While the data used in this modeling exercise were generated in the context of nutritional studies on infant formula, they may be used for any research context that can benefit from typical growth reference ranges using the domestic pig in research.

Here, we sought to answer two discrete questions. The first one was to describe typical growth in pigs used for biomedical research. In the studies analyzed, pigs were reared in an artificial setting with an alternate source of nutrition than porcine milk. Across numerous studies and husbandry practices, a simple understanding of expected body weights for a given age has not been systematically characterized. Though detailed in individual publications, immense inter-study variability makes it difficult to understand data quality and biological relevance between studies. Moreover, inter-study variability is related to the second question: What parameters most strongly affect growth? Assessing the safety of a nutritional ingredient at a given concentration requires not only the knowledge of how an experimental group fared compared to a control group, but how both groups faired compared to an existing body of knowledge. If an experimental diet (e.g., an iron-deficient diet) lowered the growth of subjects compared to a control group, but overall growth was within biological ranges, is the altered growth biologically meaningful? The reverse case could be made for a supplement trial, where improvements in metabolism might be considered modest when compared to historical references. These are a few examples of how the database presented here could contribute to provide an external context on the interpretation of individual studies.

### Toward Understanding Typical Growth

Based on the database with historical data from pig studies over time, we propose a growth percentile chart for AR pigs analogous to infant growth charts that are broadly used in pediatric practice. This reference graph facilitates easy comparison of results on animal growth from a given study with “typical” growth patterns for pigs in early life. Creation of the reference graph allowed visualizing a few striking phenomena, those being FTT, median (typical) growth, and accelerated growth. First, it became clear from pigs in the FTT group that terminally poor growth may be identifiable within the first 10 days, potentially even the first 5 days postnatal. While growth is typically slow during the first 10 days, the growth of pigs in the FTT group exhibited almost no increase in body weight growth after birth. During the first week of life both SR and AR animals displayed slow rates of growth. While poor growth is often noted by caretakers during studies and subjectively observed through metrics like body condition, the objective reference created here may allow future researchers to better identify pigs at risk of FTT and provide veterinary attention more quickly.

In addition to serving as a metric to identify terminally poor growth, the FTT group provides a reference to interpret the severity of inhibited growth. For example, pigs fed an iron deficient diet (20) showed substantially lower growth than their iron sufficient controls. However, it was not clear to what extent growth was inhibited when viewing these data without a reference. Clearly, pigs fed an iron deficient diet demonstrate reduced growth (they approximate the 25th percentile, Figure 3), however it was not as poor as to be considered FTT, and these groups did not lose body mass despite displaying signs of anemia (20). Similarly, Fleming et al. (16) describe the growth of pigs fed prebiotics as statistically lower than controls. A common difficulty of interpreting changes in body weight is understanding whether altered growth is not only “statistically different” but “biologically relevant.” When that same group was overlaid on the reference graph (Figure 3, prebiotic), it revealed that these animals grew above the 50th percentile by the end of study, suggesting that not only was growth within a typical range, but this group also slightly exceeded the median value for body weight by the end of the trial. Thus, in context of its own study (16) one might interpret the evidence to suggest that prebiotics (a combination of polydextrose and galactooligosaccharide) inhibited growth, when in reality the change in growth was within the typically observed biological range.

Conversely, it became clear that some groups grew exceptionally well. The iron sufficient group (21.3 mg/L ferrous sulfate per liter of milk), which served as an internal control for the iron deficient (2.72 mg/L) group in the same study (20) exceeded the 95th percentile by PND 30. Of note, this group was from one of three studies where pigs were fed *ad libitum* (20, 24). To our knowledge, this is the first assessment of growth outcomes between AR pigs fed *ad libitum* and in a scheduled manner. Growth curves between *ad libitum-* and scheduled-fed pigs entirely overlap until approximately PND 15 onwards, where pigs fed *ad libitum* begin to demonstrate a more rapid rate of growth (Figure 4). Thus, the iron sufficient group in Figure 3 represents an atypical case of accelerated growth.

### Rearing Environment and Sex Differences

Despite obtaining a relatively large sample size for biomedical studies (AR: *n* = 201; SR: *n* = 188), there was variability in the number of animals between studies, duration of study, and frequency of measurements both within and between the AR and SR groups, which led to highly variable mean estimates, where it appears that body weight changes significantly on a daily basis. Comparing the fitted growth models reveals that body weight variability was within 1 kg between groups at nearly all points within the first 30 days. AR pigs tended to show a slight lag in growth until ~PND 14, where their rate of growth increased, and body weight eventually surpassed that of SR pigs around PND 21 (Figure 4A). Early reports (10) suggested that pigs fed a milk replacer (PND 19–25) exhibited growth that exceeded that of suckling (i.e., sow-reared) pigs. More recent studies have shown that pigs fed milk replacer (35, 36), or even a combination of milk replacer and suckling (37), exhibit similar growth as compared to sow-reared pigs. This was shown for AR pigs fed a soy-based formula as well (36). Ultimately, the data in the present meta-analysis are supported by recent publications demonstrating similar growth between artificially- and sow-reared pigs (35–37).

Beyond growth, there were insufficient organ weight data available to compare AR and SR groups, except for intestinal weight. Both groups exhibited similar intestinal weight as a proportion of total body weight, though the AR group demonstrated higher variability than the SR group (Figure 5D). Again, this may be reflective of the data coming from multiple different studies. Practically speaking, these data suggest that power analyses for studies designed on AR or SR pigs should take these differences in variability into account for the proper estimation of sample size.

Similar to the differences between AR and SR pigs, the difference in weight between male and female pigs was <1 kg at its greatest point and was nearly identical at PND 2 and PND 30 (Figure 4B). It appeared that females exhibit numerically greater growth during the second and third weeks of life, but these differences disappeared by PND 30. This finding is supported by Yeruva et al. who reported no impact of sex on growth up to PND 21 in a study containing both SR and AR pigs (36). In sow-reared conditions, multiple reports suggest no or little effect of sex on weight at weaning. Whitley et al. (38) demonstrate that even if birth weights may differ between males and females, weaning weights (PND 24) and average daily gain between sexes are similar. Furthermore, an assessment of 8,241 pigs from Danish herds showed that males exhibited slightly lower average daily gain (4 g BW/day fewer than females) at weaning age, but this may have been related to castration (39). Practically speaking, such a difference in average daily gain over ~30 days would amount to a difference in body weight of 120 g, which most biomedical studies are not powered to detect. Taken together, most data suggest either no or a very small impact of sex on body weight. Additionally, the relative weights of the brain, intestine, and liver as a proportion of total body weight exhibited highly overlapping distributions between male and female pigs (Figures 5A–C). Thus, for purposes of identifying deviations from typical growth, stratification by sex may be unnecessary.

### Emphasizing the Value of Organ Weights as an Endpoint for Safety Assessments

To evaluate the safety of new ingredients for application in infant formula in preclinical studies, the IOM recommends a two-level assessment (1). Level 1 assessments include measures of each organ system (e.g., organ weight, histology, cell counts, etc.), with Level 2 including deeper measures of organ systems (e.g., hormones, cytokines, growth factors, etc.) required to explain findings from Level 1. Similar to understanding typical body weight growth, establishing reference values for typical organ weights will be valuable to understand organ growth. To that end, organ weight data from 158 control animals were analyzed to understand the absolute and relative (to body weight) organ weights. This allowed the detection of three scenarios (Figure 6) where the assessment of absolute and relative organ weights in a univariate manner might lead to inaccurate conclusions on the safety of dietary ingredients.

Figures 6A1,2 depicts pigs reared in a perinatal choline deficient environment compared to the reference group. Assessment of absolute brain weight indicated no difference between reference and deficient groups, while relative brain weight of the choline deficient group was greater than in the reference group. Notably, these pigs also exhibited lower body weights, thus a higher relative brain weight is more reflective of changes in body mass rather than brain growth. Indeed, when regressing body weight against relative brain weight (Figure 6A2), both the choline deficient and reference groups demonstrate similar slopes. One might be led to prematurely infer that choline deficiency impacts body weight and not brain weight. When body weight was regressed against absolute brain weight, the relationship (i.e., slope) between choline deficient and reference pigs differed (Figure 6A1). In the reference group, brain weight did not appreciably increase with body weight, due to accretion of body mass at a far higher rate than brain mass. However, during choline deficiency, animals with greater body weight demonstrated brain weights significantly higher than their smaller counterparts. The reason for this is not clear, but the relationship demonstrates that the use of absolute and relative organ weights does not accurately capture potential deviations from typical development. In other words, univariate assessments of organ weight are not sufficient to characterize typical development, whereas bivariate assessments including body mass as a predictor seem to provide a greater degree of sensitivity.

The observation that bivariate assessments of organ weight are superior to univariate is shown in two more examples. Figure 6B demonstrates the phenomenon wherein iron deficient animals displayed intestinal weights similar to the reference group, on both an absolute and relative scale. Paradoxically, the iron sufficient group used as an internal control during the study exhibited intestinal weights significantly higher than observed in the reference group. On a bivariate level, absolute intestinal weight and body weight (Figure 6B1) demonstrated a similar relationship between reference, iron deficient, and iron sufficient groups. However, the relationship between relative intestinal weight and body weight appeared markedly different between iron deficient and reference groups (Figure 6B2). The reference group exhibited a linear increase in relative intestinal weight with increasing body weight, which is reflected in a slope of nearly zero when regressing body weight against relative intestinal weight. During iron deficiency, animals with larger body weights exhibited lower relative intestinal weight, indicating that accretion of intestinal mass lagged behind accretion of total body mass.

Lastly, Figure 6C demonstrates how DEHP-fed pigs exhibit absolute and relative liver weights that appear typical when compared to the reference group, however their relationship to body mass suggests otherwise. On an absolute basis, liver weight of DEHP-fed pigs did not increase with body weight at the same rate as the reference group (Figure 6C1). On a relative basis, liver weight of DEHP-fed pigs decreased with body weight more rapidly than in reference pigs (Figure 6C2). This is perhaps one of the most striking examples where from absolute and relative liver weight alone, high exposure to DEHP might be considered safe, despite published reports that it perturbs liver weight and metabolism in rodents (40).

Organ weights serve as a first point of reference when assessing developmental immunotoxicology (41). Here, we propose a simple statistical approach to increase the value of using organ weights in the assessment of safety. By using a bivariate approach, organ and body weights may be used to more precisely understand the effect of a novel ingredient on development than either alone.

### Limitations and Future Opportunities

As mentioned throughout, there are multiple limitations that represent future opportunities for improvement of the approach and database described here. Given the evolution of methodologies and scientific objectives over the course of a decade, studies from which data was included in the database so far varied largely with respect to the study designs, sampling frequencies, and sample sizes between studies. Furthermore, it became impossible to disentangle effects of breed, source herd, nutritional composition, and feeding rates as these variables frequently co-varied with each other. These factors may have no bearing on the interpretation of data within a study as all subjects were treated similarly, but this does impact the ability to conduct or interpret future reviews and meta-analyses. Lastly, the specific data reported were all from studies conducted at a single research facility, where the authors had access to the raw data. The choice not to perform a meta-analysis and compile data from published studies from other research institutes was made deliberately to avoid issues pertaining to data ownership and intellectual property. The intention is for this database to serve as a first step toward contributions from multiple institutions, which will allow far greater power to understand biological development and the impact of additional factors on pig typical growth. A particular advantage of this approach is that data from studies that were not designed for safety or toxicological assessments can be included, helping to fill gaps in the literature where no previous safety data exist. Thus, for safety assessments of infant formula or novel ingredients for infants, the current and future iterations of the database may provide the needed reference ranges for determining safety.

## Conclusions

We compiled growth data on body and organ weights from a decade of studies using the young pig as a biomedical model. The database is the first step toward defining biological norms during critical developmental periods and reference standards that can be used to promote systematic review, meta-analysis, and use of the pig model. Specifically, it may be used as a tool for the assessment of the safety of novel ingredients used in infant nutrition.

## Data Availability Statement

The data used in this study can be found on our online GitHub repository: https://github.com/Traverse-Science/Pig-database.

## Ethics Statement

The animal study was reviewed and approved by Illinois Institutional Animal Care and Use Committee.

## Author Contributions

VV, SD, LB, QL, GG, RD, and SF: conceptualization and writing—review and editing. VV and SF: methodology, validation, and writing—original draft. VV: software, formal analysis, and visualization. SD, RD, and SF: investigation. SD and RD: resources. VV, SD, RD, and SF: data curation. SD, QL, GG, LB, SD, and RD: supervision. SF: project administration. LB: funding acquisition. All authors contributed to the article and approved the submitted version.

## Conflict of Interest

QL, GG, and LB are employees of Reckitt. VV, and SF are employees of Traverse Science. RD and SF have ownership in Traverse Science. SM is an advisor for Traverse Science. SM and RD have received grant funding from Reckitt for previous projects, but received no funding from Reckitt for this study. Reckitt provided funding to Traverse Science for conceptualization, execution, and administration of the project. Aside from project funding and employment of some of the authors, Reckitt had no other involvement in this publication.

## Publisher's Note

All claims expressed in this article are solely those of the authors and do not necessarily represent those of their affiliated organizations, or those of the publisher, the editors and the reviewers. Any product that may be evaluated in this article, or claim that may be made by its manufacturer, is not guaranteed or endorsed by the publisher.

## References

[1] Institute of Medicine. Infant Formula: Evaluating the Safety of New Ingredients. Washington, DC: The National Academies Press (2004). Available online at: https://www.nap.edu/catalog/10935/infant-formula-evaluating-the-safety-of-new-ingredients (accessed November 1, 2021).

[2] Committee ES Hardy A Benford D Halldorsson T Jeger MJ Knutsen HK. Guidance on the risk assessment of substances present in food intended for infants below 16 weeks of age. EFSA J. (2017) 15:4849. 10.2903/j.efsa.2017.4849 32625502 PMC7010120

[3] Lind NM Moustgaard A Jelsing J Vajta G Cumming P Hansen AK. The use of pigs in neuroscience: modeling brain disorders. Neurosci Biobehav Rev. (2007) 31:728–51. 10.1016/j.neubiorev.2007.02.003 17445892

[4] Odle J Lin X Jacobi SK Kim SW Stahl CH. The suckling piglet as an agrimedical model for the study of pediatric nutrition and metabolism. Annu Rev Anim Biosci. (2014) 2:419–44. 10.1146/annurev-animal-022513-114158 25384150

[5] Wang M Donovan SM. Human microbiota-associated swine: current progress and future opportunities. ILAR J. (2015) 56:63–73. 10.1093/ilar/ilv006 25991699 PMC7108572

[6] Fil JE Fleming SA Chichlowski M Gross G Berg BM Dilger RN. Evaluation of dietary bovine milk fat globule membrane supplementation on growth, serum cholesterol and lipoproteins, and neurodevelopment in the young pig. Front Pediatrics. (2019) 7:417. 10.3389/fped.2019.00417 31681715 PMC6811645

[7] Saraf MK Piccolo BD Bowlin AK Mercer KE LeRoith T Chintapalli SV. Formula diet driven microbiota shifts tryptophan metabolism from serotonin to tryptamine in neonatal porcine colon. Microbiome. (2017) 5:77. 10.1186/s40168-017-0297-z 28705171 PMC5513086

[8] Jacobi SK Yatsunenko T Li D Dasgupta S Yu RK Berg BM. Dietary isomers of sialyllactose increase ganglioside sialic acid concentrations in the corpus callosum and cerebellum and modulate the colonic microbiota of formula-fed piglets. J Nutr. (2016) 146:200–8. 10.3945/jn.115.220152 26701794

[9] Sun J Li Y Nguyen DN Mortensen MS Akker CH. van den, Skeath T, Pors SE, et al. Nutrient Fortification of human donor milk affects intestinal function and protein metabolism in preterm pigs. J Nutr. (2018) 148:336–47. 10.1093/jn/nxx033 29462356

[10] Zijlstra RT Whang KY Easter RA Odle J. Effect of feeding a milk replacer to early-weaned pigs on growth, body composition, and small intestinal morphology, compared with suckled littermates. J Anim Sci. (1996) 74:2948. 10.2527/1996.74122948x 8994909

[11] Mudd AT Dilger RN. Early-life nutrition and neurodevelopment: use of the piglet as a translational model. Adv Nutr (Bethesda, Md). (2017) 8:92–104. 10.3945/an.116.013243 28096130 PMC5227977

[12] Monaco MH Kashtanov DO Wang M Walker DC Rai D Jouni ZE. Addition of polydextrose and galactooligosaccharide to formula does not affect bacterial translocation in the neonatal piglet. J Pediatr Gastroenterol Nutr. (2011) 52:210–6. 10.1097/MPG.0b013e3181ffcaee 21240011

[13] Hoeflinger JL Kashtanov DO Cox SB Dowd SE Jouni ZE Donovan SM. Characterization of the intestinal lactobacilli community following galactooligosaccharides and polydextrose supplementation in the neonatal piglet. PLoS ONE. (2015) 10:e0135494. 10.1371/journal.pone.0135494 26275147 PMC4537252

[14] Mudd AT Waworuntu RV Berg BM Dilger RN. Dietary alpha-lipoic acid alters piglet neurodevelopment. Front Pediatr. (2016) 4:44. 10.3389/fped.2016.00044 27200325 PMC4858520

[15] Fleming SA Richards JD Bradley CL Pan X Li Q. Dilger RN. Dietary pectin at 02% in milk replacer did not inhibit growth, feed intake, or nutrient digestibility in a 3-week neonatal pig study. Regul Toxicol Pharmacol. (2020) 114:104669. 10.1016/j.yrtph.2020.104669 32360443

[16] Fleming SA Monaikul S Patsavas AJ Waworuntu RV Berg BM Dilger RN. Dietary polydextrose and galactooligosaccharide increase exploratory behavior, improve recognition memory, and alter neurochemistry in the young pig. Nutr Neurosci. (2017) 22:1–14. 10.1080/1028415X.2017.1415280 29251222

[17] Mudd AT Alexander LS Berding K Waworuntu RV Berg BM Donovan SM. Dietary prebiotics, milk fat globule membrane, and lactoferrin affects structural neurodevelopment in the young piglet. Front Pediatr. (2016) 4:4. 10.3389/fped.2016.00004 26870719 PMC4740374

[18] Mudd AT Fleming SA Labhart B Chichlowski M Berg BM Donovan SM. Dietary sialyllactose influences sialic acid concentrations in the prefrontal cortex and magnetic resonance imaging measures in corpus callosum of young pigs. Nutrients. (2017) 9:1297. 10.3390/nu9121297 29182578 PMC5748748

[19] Monaco MH Wang M Pan X Li Q Richards JD Chichlowski M. Evaluation of sialyllactose supplementation to a prebiotic-containing formula on growth, intestinal development and bacterial colonization in the neonatal piglet. Curr Dev Nutr. (2018) 2:nzy067. 10.1093/cdn/nzy067 30443641 PMC6226774

[20] Knight LC Dilger RN. Longitudinal effects of iron deficiency anemia and subsequent repletion on blood parameters and the rate and composition of growth in pigs. Nutrients. (2018) 10:632. 10.3390/nu10050632 29772815 PMC5986511

[21] Getty CM Dilger RN. Moderate perinatal choline deficiency elicits altered physiology and metabolomic profiles in the piglet. PLoS One. (2015) 10:e0133500. 10.1371/journal.pone.0133500 26196148 PMC4510435

[22] Mudd AT Alexander LS Johnson SK Getty CM Malysheva OV Caudill MA. Perinatal dietary choline deficiency in sows influences concentrations of choline metabolites, fatty acids, and amino acids in milk throughout lactation. J Nutr. (2016) 146:2216–23. 10.3945/jn.116.238832 27733523

[23] Getty CM Almeida FN Baratta AA Dilger RN. Plasma metabolomics indicates metabolic perturbations in low birth weight piglets supplemented with arginine. J Anim Sci. (2015) 93:5754–63. 10.2527/jas.2015-9293 26641185

[24] Fleming SA Chichlowski M Berg BM Donovan SM Dilger RN. Dietary sialyllactose does not influence measures of recognition memory or diurnal activity in the young pig. Nutrients. (2018) 10:395. 10.3390/nu10040395 29570610 PMC5946180

[25] Fleming SA Dilger RN. Young pigs exhibit differential exploratory behavior during novelty preference tasks in response to age, sex, and delay. Behav Brain Res. (2017) 321:50–60. 10.1016/j.bbr.2016.12.027 28042005

[26] Mudd AT Getty CM Dilger RN. Maternal dietary choline status influences brain grey and white matter development in young pigs1–3. Curr Dev Nutr. (2018) 2:nzy015. 10.1093/cdn/nzy015 29955727 PMC6007439

[27] Fil JE Joung S Zimmerman BJ Sutton BP Dilger RN. High-resolution magnetic resonance imaging-based atlases for the young and adolescent domesticated pig (*Sus scrofa*). J Neurosci Methods. (2021) 354:109107. 10.1016/j.jneumeth.2021.109107 33675840

[28] R Core Team 3,.6.2. A Language Environment for Statistical Computing. Vienna: R Foundation for Statistical Computing (2021). Available online at: https://www.r-project.org/ (accessed November 1, 2021).

[29] Wickham H Averick M Bryan J Chang W D'Agostino McGowan L Francois R. Welcome to the tidyverse. J Open Source Softw. (2019) 4:1686. 10.21105/joss.01686

[30] Fan J Gijbels I. Local Polynomial Modelling and Its Applications: Monographs on Statistics and Applied Probability. Department of Statistics, University of North Carolina Chapel Hill, NC: Routledge.

[31] Bates D Mächler M Bolker B Walker S. Fitting linear mixed-effects models using lme4. J Stat Softw. (2015) 67:1–48. 10.18637/jss.v067.i01

[32] Gueorguieva R Krystal JH. Move over ANOVA: progress in analyzing repeated-measures data andits reflection in papers published in the archives of general psychiatry. Arch Gen Psychiatry. (2004) 61:310–7. 10.1001/archpsyc.61.3.310 14993119

[33] Wickham H. ggplot2: Elegant Graphics for Data Analysis. New York, NY: Springer-Verlag. (2016). Available online at: https://ggplot2.tidyverse.org (accessed November 1, 2021).

[34] World Health Organization. Weight-for-age Percentiles Chart. Available online at: https://www.who.int/tools/child-growth-standards/standards/weight-for-age (accessed July 23, 2021)

[35] Fil JE Joung S Hayes CA Dilger RN. Influence of rearing environment on longitudinal brain development, object recognition memory, and exploratory behaviors in the domestic pig (*Sus scrofa*). Front Neurosci. (2021) 15:649536. 10.3389/fnins.2021.649536 33841090 PMC8024486

[36] Yeruva L Spencer NE Saraf MK Hennings L Bowlin AK Cleves MA. Formula diet alters small intestine morphology, microbial abundance and reduces VE-cadherin and IL-10 expression in neonatal porcine model. BMC Gastroenterol. (2016) 16:40. 10.1186/s12876-016-0456-x 27005303 PMC4804644

[37] Kobek-Kjeldager C Moustsen VA Pedersen LJ Theil PK. Impact of litter size, supplementary milk replacer and housing on the body composition of piglets from hyper-prolific sows at weaning. Animal. (2021) 15:100007. 10.1016/j.animal.2020.100007 33516024

[38] Whitley NC O'Brien DJ Quinn RW Keisler DH Walker EL Brown MA. Milk leptin in sows and blood leptin and growth of their offspring1,2. J Anim Sci. (2009) 87:1659–63. 10.2527/jas.2008-1568 19181768

[39] Johansen M Alban L Kjærsgård HD Bækbo P. Factors associated with suckling piglet average daily gain. Prev Vet Med. (2004) 63:91–102. 10.1016/j.prevetmed.2004.01.011 15099719

[40] Tassinari R Tait S Busani L Martinelli A Narciso L Valeri M. Metabolic, reproductive and thyroid effects of bis(2-ethylhexyl) phthalate (DEHP) orally administered to male and female juvenile rats at dose levels derived from children biomonitoring study. Toxicology. (2021) 449:152653. 10.1016/j.tox.2020.152653 33309551

[41] Collinge M Burns-Naas LA Chellman GJ Kawabata TT Komocsar WJ Piccotti JR. Developmental immunotoxicity (DIT) testing of pharmaceuticals: current practices, state of the science, knowledge gaps, and recommendations. J Immunotoxicol. (2012) 9:210–30. 10.3109/1547691X.2012.661486 22428536

